# Atrial Fibrillation Among ICU Patients with Type 2 Respiratory Failure: Who Is at Risk and What Are the Outcomes?

**DOI:** 10.3390/diagnostics15131612

**Published:** 2025-06-25

**Authors:** Oral Mentes, Deniz Celik, Murat Yıldız, Tarkan Özdemir, Maside Ari, Eda Nur Aksoy Güney, Emrah Ari, Fatma Canbay, Yusuf Taha Güllü, Abdullah Kahraman, Mustafa Özgür Cırık

**Affiliations:** 1 Department of Intensive Care, Gulhane Training and Research Hospital, 06010 Ankara, Turkey; omentes@live.com; 2 Department of Pulmonary Medicine, Faculty of Medicine, Alanya Alaaddin Keykubat University, 07425 Antalya, Turkey; drdenizcelik@hotmail.com; 3 Department of Pulmonary Medicine, Atatürk Sanatorium Research Hospital, Faculty of Medicine, Turkey Health Sciences University, 06830 Ankara, Turkey; masidetuten@icloud.com (M.A.); aksoyedanur95@gmail.com (E.N.A.G.); 4 Department of Pulmonology, Konya Farabi Hospital, 42090 Konya, Turkey; tarkanozdemir78@gmail.com; 5 Mamak Public Hospital, 06270 Ankara, Turkey; dremrahari25@gmail.com; 6 Department of Pulmonary Medicine, Dr. Burhan Nalbantoğlu State Hospital, 99010 Lefkoşa, Cyprus; canbayfatma5@gmail.com; 7 Anesthesia Program Mudany, Vocational School, Mudanya University, 16960 Bursa, Turkey; 8 Department of Intensive Care, Etlik City Hospital, 06170 Ankara, Turkey; abdullahhero100@gmail.com; 9 Department of Anesthesiology, Atatürk Sanatorium Research Hospital, Faculty of Medicine, Turkey Health Sciences University, 06100 Ankara, Turkey; dr.ozgurr@hotmail.com

**Keywords:** hypercapnic respiratory failure, atrial fibrillation, mortality, COPD, survival analysis

## Abstract

**Background**: Atrial fibrillation (AF) frequently occurs in individuals with hypercapnic type 2 respiratory failure and has the potential to adversely affect patient outcomes. This study sought to investigate the clinical features and prognostic significance of atrial fibrillation in patients admitted to the intensive care unit with hypercapnic type 2 respiratory failure. **Methods**: This retrospective, single-center study included 200 adult patients diagnosed with hypercapnic type 2 respiratory failure between May 2022 and May 2023. Patients were grouped according to whether atrial fibrillation was present or not. Demographic, laboratory, and echocardiographic findings, comorbidities, and outcomes were compared. Kaplan–Meier survival analysis and Cox regression were used to identify mortality predictors. **Results**: AF was present in 50.5% of patients. Those with AF were older, had higher Charlson Comorbidity Index scores, and a greater prevalence of heart failure (*p* < 0.001). No significant differences were found in arterial blood gas values. AF patients had higher urea, creatinine, and BNP levels, and lower hemoglobin, lymphocyte, eosinophil, and monocyte counts (*p* < 0.05). Echocardiography showed more severe tricuspid and mitral regurgitation, lower ejection fractions, and higher systolic pulmonary pressures in the AF group. About 20% of AF patients were not receiving anticoagulants at ICU admission. AF was associated with shorter survival (49.6 ± 4.07 vs. 61.4 ± 3.8 days, *p* = 0.031) and 1.6-fold higher mortality risk (HR: 1.60, 95% CI: 1.04–2.47). Advanced age and low hemoglobin were independent predictors of mortality. **Conclusions**: AF is frequent among patients with type 2 respiratory failure and is linked to increased mortality. Despite known complications, treatment remains underutilized. AF should be actively screened during ICU admissions for respiratory failure.

## 1. Introduction

Hypercapnic respiratory failure is a systemic syndrome marked by elevated partial carbon dioxide (pCO_2_) levels due to hypoventilation, commonly seen in chronic respiratory disorders such as chronic obstructive pulmonary disease (COPD). Respiratory failure can directly impact patient survival due to inadequate oxygenation and hemodynamic alterations resulting from hypercapnia [1,2]. Hypercapnic respiratory failure extends beyond the respiratory system and may result in cardiovascular complications. Atrial fibrillation (AF) is a frequent rhythmic condition observed in these patients and significantly impacts patient prognosis.

Analysis of the pathophysiology of atrial fibrillation revealed that hypoxia and hypercapnia are significant factors. In hypercapnic individuals, factors such as intrathoracic pressure fluctuations, alterations in autonomic nervous system activity, and atrial stretching and remodeling contribute to the onset of AF [3]. Moreover, hypercapnia and hypoxemia can induce right ventricular hypertension and right atrial dilation by increasing pulmonary arterial pressure. These alterations result in cardiac hemodynamic imbalances, promoting the onset of atrial fibrillation. This may not only increase the incidence of atrial fibrillation but also negatively impact clinical outcomes, including cardioversion efficacy, ablation results, and in-hospital mortality [4].

In several patients with hypercapnic respiratory failure monitored in pulmonary intensive care units, concurrent atrial fibrillation has been noted to be associated with a poor prognosis. AF may worsen respiratory function problems, impair cardiopulmonary balance, and make therapeutic procedures more difficult for this patient population. The frequency of AF in individuals with COPD has been reported to be twice as high as that in those without COPD. Moreover, atrial fibrillation is associated with an elevated risk of mortality in this patient population [5].

The presence of AF in patients with hypercapnic respiratory failure is often accompanied by additional clinical risks, such as heart failure. Furthermore, alterations in echocardiographic findings and the increased relevance of certain laboratory parameters are inevitable in this context. Identifying the clinical profiles of patients with type 2 respiratory failure who also have AF is of great importance. Recognizing these profiles can facilitate earlier diagnosis and help determine which patients should undergo more frequent ECG screening for timely detection of arrhythmias [6,7].

Considering the well-known severe complications of AF—such as acute coronary syndrome, heart failure, valvular diseases, and ischemic cerebrovascular events—demonstrating its potential additive negative impact on mortality in this patient population would provide a meaningful contribution to the medical literature.

This study investigated the impact of AF on survival in patients with hypercapnic type 2 respiratory failure in a multidimensional manner. This study specifically investigated how pulmonary hypertension, cardiac hemodynamics, and blood gas changes affect the onset of AF in this patient population. Additionally, the impact of AF on clinical outcomes such as diagnosis, treatment, and in-hospital mortality will be assessed. The findings of this study may aid in the formulation of clinical treatment plans for this diverse patient population.

## 2. Materials and Methods

The study was carried out in compliance with institutional and national ethical guidelines, as well as the principles of the 1964 Declaration of Helsinki and its subsequent revisions. Informed consent was obtained from all participants involved in the study. Consent to publish the study findings was also obtained from all participants. In our retrospective studies, during the process of obtaining informed consent regarding procedures to be performed in the intensive care unit, patients and/or their legal representatives are asked to sign a “Retrospective Data Use Consent Form.” This form ensures that privacy regulations will be followed and authorizes the use of anonymized demographic data, laboratory results, clinical scores, ECGs, chest X-rays, and other relevant examinations for research purposes. Patients who explicitly refused permission by signing the form or who left the consent form unsigned were excluded from the study.

**Figure 1:**
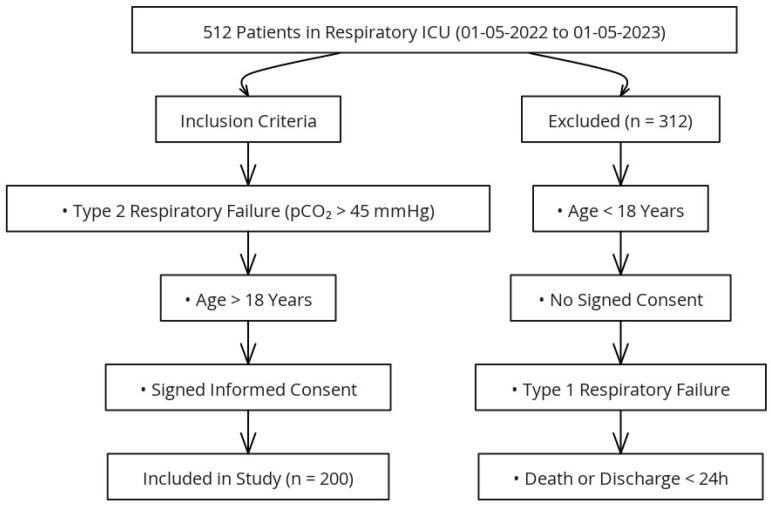
Flowchart of the study design.

This study was approved by the Clinical Research Ethics Committee of the University of Health Sciences, Ankara Sanatorium Training and Research Hospital (Date: 12 June 2024, Decision No: 2024-BÇEK/81). The study group consisted of 512 patients who were followed up in the respiratory intensive care unit between 1 May 2022 and 1 May 2023. Among these, 200 patients diagnosed with type 2 respiratory failure meeting the inclusion criteria were included in the study, while patients under the age of 18, those with incomplete medical records, patients with type 1 respiratory failure, and those referred to other centers were excluded (Figure 1).

All relevant data—such as age, sex, comorbidities, laboratory values, Charlson Comorbidity Index (CCI), and other demographic and clinical information—were retrieved from patient records and the hospital’s information management system. The diagnosis of “respiratory failure” was confirmed through arterial blood gas (ABG) values obtained during hospitalization and follow-up. AF was diagnosed based on electrocardiographic (ECG) findings obtained at ICU admission, which is routinely performed for all patients. Patients were classified as having AF if the arrhythmia was present on admission ECG and persisted throughout the ICU stay. Individuals with paroxysmal AF—defined as AF episodes that spontaneously resolve or terminate with intervention within 7 days—were excluded. Accordingly, patients with chronic or persistent AF were included in the AF group. Due to the retrospective nature of the study, a more granular distinction between persistent and chronic AF was not possible. Echocardiographic findings within the last year and the use of antiarrhythmic and anticoagulant drugs were reviewed.

Patients were stratified into two groups based on the presence or absence of AF. Blood parameters routinely analyzed during intensive care follow-up were retrospectively examined. The blood parameters were analyzed comparatively between the two groups to assess their impact on in-hospital mortality and length of stay.

### Statistical Analysis

Statistical analyses were conducted using IBM SPSS Statistics for Windows, Version 30.0 (IBM Corp., Armonk, NY, USA) and MedCalc version 23.2.1 (MedCalc Software Ltd., Ostend, Belgium). The distribution of continuous variables was evaluated through descriptive measures, including the Kolmogorov–Smirnov and Shapiro–Wilk tests, skewness–kurtosis values, histograms, and outlier inspection. Normally distributed data were expressed as means ± standard deviations, whereas non-normally distributed data were presented as medians with interquartile ranges (IQR). Categorical variables were reported as frequencies and percentages.

Comparisons between categorical variables were performed using Pearson’s chi-square test, and Fisher’s exact test was applied when expected cell counts were below 5. For continuous variables, Student’s *t*-test was used when data met normality assumptions, while the Mann-Whitney U test was applied for non-normally distributed variables. Survival outcomes were assessed using Kaplan–Meier analysis, with the log-rank test employed to determine significance. Cox proportional hazards regression analysis was performed to identify independent predictors of mortality, and hazard ratios (HRs) with 95% confidence intervals (CIs) were reported. A two-tailed ***p***-value < 0.05 was considered statistically significant.

## 3. Results

The study was conducted with 200 patients who met the eligibility criteria. Among the patients, 118 (59%) were male. The mean age of the patients was 72 ± 9 years. The most common comorbidity was chronic obstructive pulmonary disease (COPD). The demographic and clinical characteristics of the patients are presented in Table 1.

When the demographic characteristics of patients were evaluated on the basis of the presence of AF, patients with atrial fibrillation were older (*p* < 0.001). An examination of comorbid conditions revealed that heart failure was more common and that the CCI was greater in these patients (*p* < 0.001). Additionally, mortality was found to be greater in patients with AF (*p* = 0.043) (Table 2).

When patients with type 2 respiratory failure with AF were compared to those without AF based on arterial blood gas values at ICU admission, no statistically significant difference was found. The admission blood gas values of patients who were followed up with hypercapnic respiratory failure are presented in Table 3.

AF was present in 50.5% of the patients included in the study. The blood test results of the patients based on the presence of AF are presented in Table 4. Urea and creatinine levels were found to be higher in patients with AF (*p* < 0.001). Among the complete blood count parameters, the hemoglobin, lymphocyte, eosinophil, and monocyte levels were lower in patients with AF (*p* < 0.001, *p* = 0.004, *p* = 0.029, *p* = 0.047, respectively). On the other hand, natriuretic peptide levels were greater in patients with AF (*p* < 0.001) (Table 4).

Among the patients included in the study, 42.5% passed away within 30 days of follow-up. The admission blood test results of the patients were evaluated on the basis of overall averages and mortality status. In deceased patients, urea, brain natriuretic peptide (BNP), troponin, and D-dimer levels were significantly greater (*p* = 0.046, *p* = 0.017, *p* = 0.02, *p* = 0.011, respectively). Hemoglobin levels, on the other hand, were lower in deceased patients (*p* = 0.06) (Table 5). When we compared the categorical variables in our study in terms of mortality, we found that mortality was significantly higher in patients with heart failure and atrial fibrillation (Table 6).

A total of 125 patients underwent echocardiographic evaluation, of whom 65 had AF and 60 did not. Upon evaluating the echocardiographic findings of patients admitted to our intensive care unit with a diagnosis of type 2 respiratory failure, we identified certain statistically significant differences between those with and without AF. Patients with AF had more severe tricuspid and mitral regurgitation (*p*: 0.0047 and *p*: 0.0135, respectively). Ejection fraction values were also significantly lower in patients with AF (*p*: 0.001). Additionally, systolic pulmonary arterial pressures were significantly higher in patients with AF (*p*: 0.016). However, there was no statistically significant difference in the prevalence of aortic regurgitation between the two groups (Table 7).

To analyze the association of atrial fibrillation (AF) with other clinical conditions and comorbidities, the prevalence of additional diseases was examined in detail among patients with and without AF. Heart failure was the only comorbidity found to be significantly more common in patients with AF (*p* < 0.001). All other clinical conditions and comorbidities were statistically similar between the two groups (Table 8).

Among patients admitted to the intensive care unit with pre-existing atrial fibrillation (AF), 19.8% were not prescribed anticoagulant therapy and 6.9% were not receiving antiarrhythmic medications at the time of admission (Table 9).

**Figure 2:**
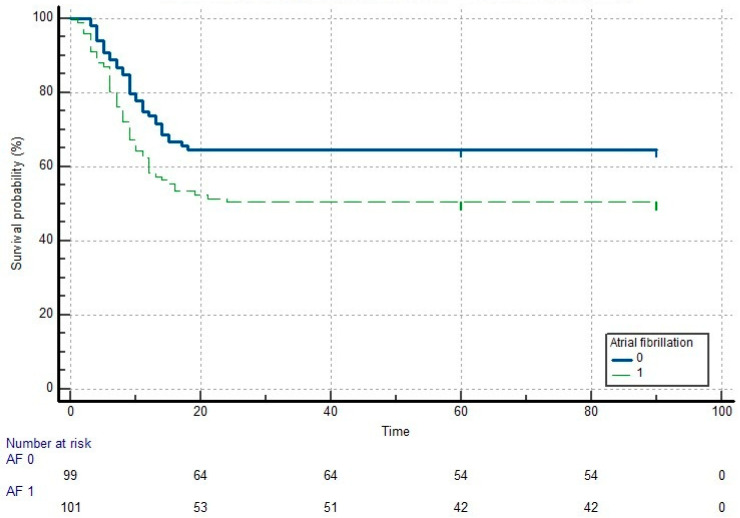
Kaplan-Meier survival curve for patients with and without atrial fibrillation.

When comparing the survival times of patients with and without atrial fibrillation (AF), the mean survival time was found to be 49.6 (±4.07) days (95% CI: 41.6 to 57.6) for patients with AF and 61.4 (±3.8) days (95% CI: 53.8 to 69.05) for those without AF. The difference in survival times was statistically significant, favoring patients without AF (log-rank test: *p* = 0.031). Furthermore, according to this analysis, the mortality risk in patients with AF was calculated to be 1.6 times higher compared to those without AF (HR: 1.6084, 95% CI: 1.0437 to 2.4787) (Figure 2).

A multivariable Cox regression analysis was performed using the enter method, including all variables that were significantly associated with mortality in univariate analyses. Among these, lower hemoglobin levels (HR: 0.901; 95% CI: 0.816–0.996; *p* = 0.041), older age (HR: 1.039; 95% CI: 1.007–1.073; *p* = 0.018), and higher Charlson Comorbidity Index scores (HR: 1.175; 95% CI: 1.004–1.375; *p* = 0.045) were found to be independent predictors of in-hospital mortality. Notably, the presence of atrial fibrillation (HR: 0.883; 95% CI: 0.532–1.467; *p* = 0.631) did not independently predict mortality in this model (Table 10).

## 4. Discussion

### 4.1. Prevalence and Clinical Characteristics of AF in Type 2 Respiratory Failure

One of the most prominent findings of our study was the detection of AF in half of the patients admitted to our respiratory intensive care unit with a diagnosis of type 2 respiratory failure over a one-year period. In the literature, AF is recognized as the most common arrhythmia accompanying patients with type 2 respiratory failure and COPD [8]. When we compared the clinical characteristics of patients with AF to those without AF among individuals with type 2 respiratory failure, we observed that patients with AF were older, had higher CCI scores—indicating a greater burden of comorbidities—and had a notably higher prevalence of heart failure. In parallel with our findings, the study conducted by Chen and Liao also demonstrated that patients with AF were older and had a higher prevalence of cardiac comorbidities, including heart failure, compared to those without AF [9].

### 4.2. Laboratory Abnormalities and Their Pathophysiological Implications

When further examining the characteristics of patients with AF in our study through laboratory test results, we observed that urea and creatinine levels were higher in patients with AF compared to those without AF and that natriuretic peptide levels were also elevated, consistent with the higher prevalence of heart failure in this group. Additionally, hemoglobin and lymphocyte levels were found to be lower in patients with AF. Rodríguez-Manero et al. reported elevated natriuretic peptide levels in patients with AF, attributing this to the high prevalence of heart failure in this population. Similarly, Terzano et al. found that urea and creatinine levels were higher in AF patients, explaining this finding in the context of heart failure and associated systemic hypoperfusion. Chen and Liao not only noted that anemia was more common in patients with AF, but—consistent with our findings—also emphasized its association with poor prognosis in this group. Furthermore, Romiti et al. suggested that lower lymphocyte counts observed in patients with AF reflect systemic inflammation and a weakened immune response [8,9,10,11]. These laboratory abnormalities in our AF cohort may reflect a more advanced stage of cardio-respiratory compromise, particularly in the context of chronic hypercapnic respiratory failure. In diseases that typically cause type 2 respiratory failure—such as COPD and obesity hypoventilation syndrome—chronic hypoventilation and right heart strain can exacerbate renal hypoperfusion and systemic inflammation, amplifying neurohormonal activation. Elevated urea and BNP levels may therefore reflect not only cardiac dysfunction but also an adaptive response to chronic hypercapnia and fluid retention. Similarly, the observed anemia and lymphopenia may be consequences of systemic inflammation, reduced erythropoietin activity, and nutritional deficiencies common in chronic respiratory insufficiency. These findings suggest that the laboratory profile of AF patients in type 2 respiratory failure represents a complex interplay between cardiovascular strain, respiratory mechanics, and systemic inflammation.

### 4.3. Echocardiographic Features Suggestive of Cardiac Dysfunction

When comparing the echocardiographic findings of our AF patients to those without AF among individuals with hypercapnic respiratory failure, we observed that advanced mitral and tricuspid regurgitation were more common in the AF group. Additionally, systolic pulmonary arterial pressures were higher, and ejection fraction values were lower in patients with AF. These findings suggest that AF in the context of hypercapnic respiratory failure is associated with more severe underlying cardiac dysfunction. Similarly, Terzano et al. reported that patients with COPD exacerbations and AF had significantly higher pulmonary artery pressures and a greater incidence of valvular abnormalities, particularly mitral regurgitation. Reduced ejection fraction was also more common in the AF group, highlighting the interplay between AF and impaired cardiac performance in respiratory patients [5,11].

### 4.4. AF and Mortality: Interpretation of Survival Analyses

Based on our survival analysis, we observed that patients with AF had shorter survival times compared to those without AF among individuals admitted to the intensive care unit with type 2 respiratory failure. However, in the enter method Cox regression analysis—which included the parameters that differed significantly between survivors and non-survivors—AF did not emerge as an independent predictor of mortality [12]. One possible explanation is that AF may function more as a surrogate marker of systemic burden rather than as a direct contributor to mortality. In critically ill patients, particularly those with advanced age and multiple comorbidities, AF often reflects underlying physiological stress, inflammation, or cardiac dysfunction. When variables such as age and Charlson Comorbidity Index are accounted for, the additive prognostic value of AF appears to diminish. Furthermore, in the context of acute-on-chronic respiratory failure, factors such as gas exchange impairment and baseline anemia may exert a more immediate impact on outcomes, overshadowing the contribution of arrhythmias. These findings suggest that while AF may signal increased clinical complexity, it may not independently drive mortality risk in this specific ICU population. This finding aligns with results from Rodríguez-Mañero et al., who also found that while AF was associated with worse unadjusted survival in patients with COPD, it did not independently predict mortality after adjusting for age and comorbidities, emphasizing the stronger prognostic weight of systemic factors such as age and overall disease burden [10]. Similarly, Xiao et al. reported that although AF prevalence was high in end-stage COPD patients, mortality was more strongly influenced by age, need for mechanical ventilation, and comorbidity burden rather than AF itself in multivariable analyses [13]. Considering the contribution of low hemoglobin levels to mortality in type 2 respiratory failure, future studies may focus on this issue to determine whether a new transfusion threshold should be established specifically for this patient population. Just as the presence of cardiac disease can raise the transfusion threshold in intensive care units, type 2 respiratory failure itself—independent of cardiac comorbidities—may warrant a reassessment of red blood cell replacement criteria [14].

### 4.5. Gas Exchange Parameters and AF

In our study, there were no statistically significant differences in pCO_2_, pO_2_, pH, or HCO_3_ levels between patients with and without AF at the time of ICU admission. This suggests that the presence of AF may not be directly related to the severity of gas exchange abnormalities at presentation. Supporting this, Lahousse et al. found that while reduced lung function was associated with increased AF risk over time, cross-sectional arterial blood gas values—such as pCO_2_ and pH—did not differ significantly at baseline between patients who developed AF and those who did not, emphasizing the role of chronic pulmonary and cardiovascular remodeling over acute respiratory derangement in AF pathophysiology [15].

### 4.6. Inadequate Pre-ICU AF Management and Therapy Gaps

In our study, we identified significant gaps in pre-ICU management by comparing AF diagnoses—based on ECGs obtained at ICU admission—with the patients’ ongoing anticoagulant and antiarrhythmic therapies at the time of admission. These deficiencies were particularly notable in anticoagulant therapy, which is vital for preventing thrombotic complications. Despite having a diagnosis of AF, approximately 20% of patients were not receiving any anticoagulant treatment prior to ICU admission, while about 7% were not on antiarrhythmic therapy [16]. These findings are in line with the results of Wang et al., who reported substantial underutilization of anticoagulants in patients with AF, especially among those with chronic comorbidities such as COPD. Their study highlighted that up to one-quarter of eligible AF patients were not prescribed anticoagulants, often due to concerns about bleeding risk or a lack of cardiology follow-up, reflecting a broader issue of suboptimal adherence to evidence-based AF management in high-risk populations [17]. We share and support this perspective, as our findings similarly highlight a disconnect between AF diagnosis and the implementation of guideline-directed therapies. The underuse of anticoagulation observed in our cohort—despite a clear clinical indication—underscores the need for improved coordination between outpatient cardiology and ICU teams. It also points to the importance of early medication reconciliation and intervention planning at the point of ICU admission, particularly in patients with complex chronic diseases such as COPD.

### 4.7. Clinical Implications and Need for Multidisciplinary Care

Taken together, our findings highlight the multifactorial nature of AF in patients with type 2 respiratory failure, particularly within the intensive care setting. The coexistence of AF with elevated age, comorbidity burden, and cardiac dysfunction reflects a complex pathophysiological interaction rather than a direct effect of respiratory acidosis or gas exchange parameters. This complexity has been echoed in prior literature, where systemic inflammation, ventricular strain, and impaired myocardial oxygenation are increasingly recognized as key contributors to AF onset and progression in COPD and ICU cohorts. Furthermore, persistent gaps in the application of guideline-directed anticoagulation and rhythm control therapies suggest a real-world treatment inertia that may negatively impact outcomes. These findings underscore the need for a multidisciplinary approach to AF management in critically ill respiratory patients, incorporating early cardiology consultation, comprehensive risk stratification, and improved adherence to evidence-based therapies [18,19,20].

### 4.8. Limitations of the Study

This study presents several limitations. Primarily, its retrospective and single-center design may restrict the generalizability of the results to broader populations. Additionally, the diagnosis of atrial fibrillation was based solely on ECG recordings obtained at ICU admission, and paroxysmal AF cases may have been missed. Echocardiographic evaluations were not available for all patients and were conducted only in those with accessible records; therefore, cardiac functional data do not represent the entire study population. Furthermore, data on anticoagulant and antiarrhythmic therapy were extracted from hospital records, without access to detailed information regarding treatment adherence or reasons for discontinuation. Lastly, the absence of long-term follow-up data limits our ability to evaluate post-discharge outcomes and the long-term impact of atrial fibrillation on morbidity and mortality.

## 5. Conclusions

This study demonstrated that atrial fibrillation (AF) is highly prevalent among patients admitted to the intensive care unit with a diagnosis of type 2 respiratory failure. AF in this population was associated with older age, a greater burden of comorbidities, higher rates of heart failure, and abnormalities in both hematologic and cardiac biomarkers. Although AF appeared to be associated with increased mortality in univariate survival analyses, it did not emerge as an independent predictor in multivariable models. Instead, age and hemoglobin levels were identified as independent determinants of mortality. Our findings also revealed a noteworthy proportion of patients with AF who were not receiving appropriate anticoagulant therapy. Early recognition of AF, identification of cardiac dysfunction, and timely implementation of appropriate treatment strategies are crucial to improving prognosis in this patient group. In this context, multidisciplinary evaluation and future prospective studies with larger cohorts are warranted to enhance clinical management and outcomes.

## Figures and Tables

**Table 1:** Demographic and Clinical Characteristics of All Patients.

Variable	Value—*n* (%), Mean (±sd), Median (IQR)
Age, years	72 ± 9
Presence of atrial fibrillation	101 (50.5%)
Mortality status	85 (42.5%)
Gender	
Male	118 (59%)
Female	82 (41%)
Comorbidity	
Chronic obstructive pulmonary disease	169 (84.5%)
Hypertension	103 (51.5%)
Coronary artery disease	26 (13%)
Diabetes mellitus	77 (38.5%)
Chronic kidney disease	24 (12%)
Bronchiectasis	8 (4%)
Malignancy	8 (4%)
Obesity hypoventilation syndrome	6 (3%)
Past tuberculosis enfection	7 (3.5%)
Dementia	8 (4%)
Acute Kidney Failure	5 (2.5%)
Past cerebrovascular disease	11 (5.5%)
Pulmonary embolism	8 (4%)
Heart failure	68 (34%)
Charlson comorbidity index	5 (4–7)
Length of hospital stay	8.94 ± 5.24

**Table 2:** Demographic characteristics of patients based on the presence of atrial fibrillation.

Demographic Characteristics	Patients with AF (*n* = 101)	Patient Without AF (*n* = 99)	*p* Value
Age, years (Mean ± sd)	76 ± 8	69 ± 8	<0.001 *^α^
Gender			0.059 ^β^
Male *n* (%)	53 (%44.9)	65 (%55.1)	
Female *n* (%)	48 (%58.5)	34 (%41.5)	
Number of deceased patients *n* (%)	59 (%58.4)	45 (%45.4)	0.043 *^β^
Comorbidity			
COPD *n* (%)	81 (%47.9)	88 (%52.1)	0.090 ^β^
HT *n* (%)	53 (%51.5)	50 (%48.5)	0.781 ^β^
DM *n* (%)	39 (%50.6)	38 (%49.4)	0.973 ^β^
HF *n* (%)	46 (%67.6)	22 (%32.4)	<0.001 *^β^
CCI median (IQR)	6 (4–7)	4 (4–6)	<0.001 *^γ^
ICU length of stay (Mean ± sd)	9.08 ± 6.29	8.79 ± 3.92	0.829 ^α^

AF: Atrial Fibrillation; CCI: Charlson Comorbidity Index; COPD: chronic obstructive pulmonary disease; HT: hypertension; ICU: intensive care unit; ^α^: Student’s *t* test; ^β^: chi-square test; ^γ^: Mann-Whitney U test; *: statistically significant values.

**Table 3:** The admission blood gas values of patients.

Admission Blood Gas	Patients with AF (*n* = 101) Median (IQR)	Patients Without AF (*n* = 99) Median (IQR)	*p* Value
pH	7.33 (7.24–7.42)	7.33 (7.29–7.39)	0.754 ^γ^
paO_2_	50 (32.2–72.7)	48 (33–62)	0.422 ^γ^
pCO_2_	66 (56–83)	67 (59–78)	0.835 ^γ^
HCO_3_	36 (32.2–42)	37 (32–42)	0.607 ^γ^

AF: Atrial fibrillation; ^γ^: Mann-Whitney U test.

**Table 4:** Evaluation of Patient Admission Blood Tests.

Laboratory Findings	Patient with AF (*n* = 101) Median (IQR)	Patient Without AF (*n* = 99) Median (IQR)	*p* Value
Urea (mg/dL)	58 (41.5–82.2)	47.5 (29.2–64.5)	<0.001 *^γ^
Creatinine (mg/dL)	1.07 (0.89–1.37)	0.96 (0.77–1.29)	0.014 *^γ^
Hemoglobin (g/dL)	11.05 (9.6–13.5)	12.9 (11.2–14.6)	<0.001 *^γ^
Leukocyte (×10^3^/µL)	9.8 (7.9–13.05)	9.6 (7.9–14.5)	0.301 ^γ^
Lymphocyte (×10^3^/µL)	0.86 (0.55–1.4)	1.09 (0.66–1.6)	0.004 *^γ^
Neutrophil (×10^3^/µL)	8.03 (5.7–10.9)	7.75 (5.7–11.7)	0.967
Platelet (×10^3^/µL)	235 (174–313)	245 (191–315)	0.537 ^γ^
Eosinophil (×10^3^/µL)	0.02 (0–0.1)	0.025 (0–0.11)	0.029 *^γ^
Monocyte (×10^3^/µL)	0.57 (0.32–0.76)	0.62 (0.37–0.82)	0.047 *^γ^
Natriuretic Peptide	358 (150–910)	100 (41–280)	<0.001 *^γ^
D-dimer	1645 (617–3287)	1315 (592–2455)	0.466 ^γ^
Troponin	17 (8–35)	13 (5.2–35.5)	0.156 ^γ^

^γ^: Mann-Whitney U test, *: Statistically significant values.

**Table 5:** Evaluation of Patient Admission Blood Tests on the basis of mortality.

Laboratory Findings	Surviving Patients (*n*: 115) Median (IQR)	Deceased Patients (*n*: 85) Median (IQR)	*p* Value
Urea (mg/dL)	51.00 (32.25–66.00)	57.50 (40.00–82.25)	0.046 *^γ^
Creatinine (mg/dL)	0.98 (0.80–1.20)	1.07 (0.84–1.39)	0.341 ^γ^
Potassium (mEq/L)	4.50 (4.20–4.88)	4.60 (4.20–5.20)	0.115 ^γ^
Hemoglobin (g/dL)	12.80 (11.03–14.55)	11.25 (9.58–13.50)	0.006 *^γ^
WBC (×10^3^/µL)	9.8 (7.8–13.5)	9.6 (8–13.8)	0.651 ^γ^
Lymphocyte (×10^3^/µL)	0.94 (0.63–1.5)	0.92 (0.64–1.5)	0.670 ^γ^
Platelet (×10^3^/µL)	246.5 (198–311)	233 (167–328)	0.887 ^γ^
Magnesium (mg/dL)	2.00 (1.80–2.20)	2.00(1.80–2.30)	0.103 ^γ^
Natriuretic Peptide (ng/L)	158.50 (61.75–414.00)	279.50 (112.00–855.00)	0.017 *^γ^
D-dimer (ng/mL)	940 (552–2112)	1820 (940–3755)	0.002 *^γ^
Troponin I (ng/mL)	12 (5.23–27.25)	19.50 (10–36.5)	0.011 *^γ^
Age	70.2 ± 0.87	76.2 ± 0.93	<0.001
CCI	4.50 (4.0–6.0)	5.00 (4.0–7.0)	<0.001
pH	7.35 (7.27–7.41)	7.32 (7.26–7.39)	0.324
PO2	50.00 (33.8–72.0)	45.00 (31.5–66.0)	0.434
PCO2	66.00 (57.0–81.0)	66.00 (58.5–81.0)	0.641
HCO3	37.00 (33.0–42.0)	34.00 (31.0–42.5)	0.262
Monocyte count (×10^3^/µL)	0.56 (0.34–0.76)	0.3 (0.11–0.61)	0.773
ICU Length of Stay (days)	90.00 (90.0–90.0)	8.00 (5.0–12.0)	<0.001

CCI: Charlson comorbidity index, ICU: Intensive care unit, WBC: White blood cell, ^γ^: Mann-Whitney U test, *: Statistically significant values.

**Table 6:** Comparison of categorical variables between deceased and surviving patients using the Pearson chi-square test.

Variable	Deceased (*n*, %)	Survived (*n*, %)	*p*-Value
Gender (Male)	51 (43.2%)	67 (56.8%)	0.805
Gender (Female)	34 (41.5%)	48 (58.5%)	
NIMV Required	48 (41.4%)	68 (58.6%)	0.706
NIMV Not Required	37 (44.0%)	47 (56.0%)	
Anticoagulant Use (Yes)	46 (47.9%)	50 (52.1%)	0.137
Anticoagulant Use (No)	39 (37.5%)	65 (62.5%)	
Antiarrhythmic Use (Yes)	68 (44.2%)	86 (55.8%)	0.386
Antiarrhythmic Use (No)	17 (37.0%)	29 (63.0%)	
Atrial Fibrillation (Yes)	50 (49.5%)	51 (50.5%)	0.043 *
Atrial Fibrillation (No)	35 (35.4%)	64 (64.6%)	
Heart Failure (Yes)	36 (52.9%)	32 (47.1%)	0.032 *
Heart Failure (No)	49 (37.1%)	83 (62.9%)	
Diabetes Mellitus (Yes)	32 (41.6%)	45 (58.4%)	0.831
Diabetes Mellitus (No)	53 (43.1%)	70 (56.9%)	
Hypertension (Yes)	42 (40.8%)	61 (59.2%)	0.611
Hypertension (No)	43 (44.3%)	54 (55.7%)	
COPD (Yes)	72 (42.6%)	97 (57.4%)	0.945
COPD (No)	13 (41.9%)	18 (58.1%)	

COPD: Chronic obstructive pulmonary disease, NIMV: Noninvasive mechanic ventilation, * significant *p* values.

**Table 7:** Comparison of Echocardiographic Findings Based on Atrial Fibrillation Status.

Variable	AF Negative(−) Patients *n* (%), Median (IQR), *n*: 60	AF Positive (+) Patients *n* (%), Median (IQR), *n*: 65	*p* Value
Tricuspid Regurgitation	1: 31 (51.7%) 2: 26 (43.3%) 3: 3 (5%)	1: 23 (35.4%) 2: 25 (38.5%) 3: 17 (26.2%)	0.0047 *^ɵ^
Mitral Regurgitation	1: 53 (88.3%) 2: 6 (10.0%) 3: 1 (1.7%)	1: 43 (66.2%) 2: 19 (29.2%) 3: 3 (4.6%)	0.0135 *^ɵ^
Aortic Regurgitation	1: 58 (96.7%) 2: 2 (3.3%)	1: 61 (93.8%) 2: 4 (6.2%)	0.4629 ^ɵ^
SPAP (mmHg)	40.0 (33.25–47.75)	45.0 (35.0–52.5)	0.016 *^γ^
Ejection fractio*n* (%)	55 (55–60)	55 (45–60)	0.001

Regurgitation grades are defined as follows: 1 = mild, 2 = moderate, 3 = severe. SPAP: Systolic pulmonary arterial pressure. ^ɵ^ Fisher’s exact test, ^γ^ Mann–Whitney U test, * significant *p* value.

**Table 8:** Comparison of clinical conditions between AF (+) and AF (−) patients.

Clinical Condition	AF (−) *n*: 99 *n* (%)	AF (+) *n*: 101 *n* (%)	*p* Value
Female Gender	34 (34.3%)	48 (47.5%)	0.058 ^β^
Asthma	2 (2.0%)	5 (5.0%)	0.260 ^ɵ^
OSAS	3 (3.0%)	3 (3.0%)	0.980 ^ɵ^
Malignancy	2 (2.0%)	6 (5.9%)	0.157 ^ɵ^
COPD	88 (88.9%)	81 (80.2%)	0.090 ^β^
Bronchiectasis	5 (5.1%)	3 (3.0%)	0.453 ^ɵ^
History of TB	2 (2.0%)	5 (5.0%)	0.260 ^ɵ^
Dementia/Alzheimer	2 (2.0%)	6 (5.9%)	0.157 ^ɵ^
Heart Failure	22 (22.2%)	46 (45.5%)	<0.001 *^β^
Chronic Kidney Disease	10 (10.1%)	14 (13.9%)	0.413 ^β^
Pulmonary Thromboembolism	3 (3.0%)	5 (5.0%)	0.488 ^ɵ^
Coronary Artery Disease	13 (13.1%)	13 (12.9%)	0.956 ^β^
Hypertension	50 (50.5%)	53 (52.5%)	0.780 ^β^
Diabetes Mellitus	38 (38.4%)	39 (38.6%)	0.973 ^β^
Acute Kidney Injury	1 (1.0%)	4 (4.0%)	0.181 ^ɵ^
History of Stroke	6 (6.1%)	5 (5.0%)	0.731 ^β^

COPD: Chronic obstructive pulmonary disease, TB: Tuberculosis, ^β^ Pearson chi square test, ^ɵ^ Fisher’s exact test, * significant *p* value.

**Table 9:** AF (+) patients without prescribed anticoagulants or antiarrhythmics at ICU admission.

Treatment Type	Not Prescribed (*n*)	Total AF (+) Patients (*n*)	Rate (%)
Anticoagulant	20	101	19.8%
Antiarrhythmic	7	101	6.9%

**Table 10:** Enter method Cox regression analysis results of all patients.

Variable	*p* Value	HR (Exp (B))	95% CI Lower	95% CI Upper
Urea	0.435	0.997	0.991	1.004
Hemoglobin	0.041*	0.901	0.816	0.996
Natriuretic peptide	0.122	1.0	1.0	1.0
D-Dimer	0.661	1.0	1.0	1.0
Troponine	0.293	1.0	1.0	1.001
Presence of AF	0.631	0.883	0.532	1.467
Age	0.018 *	1.039	1.007	1.073
CCI	0.045 *	1.175	1.004	1.375

AF: Atrial fibrillation, CCI: Charlson comorbidity index, HR: Hazard ratio, CI: Confidence interval, * significant *p* value.

## Data Availability

The datasets used and/or analyzed during the current study are available from the corresponding author on reasonable request.
